# Plant-Based Protein Bioinks with Transglutaminase Crosslinking: 3D Printability and Molecular Insights from NMR and Synchrotron-FTIR

**DOI:** 10.3390/foods15020322

**Published:** 2026-01-15

**Authors:** Jaksuma Pongsetkul, Sarayut Watchasit, Tanyamon Petcharat, Marcellus Arnold, Yolanda Victoria Rajagukguk, Passakorn Kingwascharapong, Supatra Karnjanapratum, Pimonpan Kaewprachu, Lutz Grossmann, Young Hoon Jung, Saroat Rawdkuen, Samart Sai-Ut

**Affiliations:** 1 School of Animal Technology and Innovation, Institute of Agricultural Technology, Suranaree University of Technology, Nakhon Ratchasima 30000, Thailand; jaksuma@sut.ac.th; 2 Department of Chemistry, Faculty of Science, Burapha University, Chonburi 20131, Thailand; sarayut.wa@buu.ac.th; 3 Professional Culinary Arts Program, School of Management, Walailak University, Nakhon Si Thammarat 80161, Thailand; tanyamon.pe@wu.ac.th; 4 Department of Gastronomy Science and Functional Foods, Faculty of Food Science and Nutrition, Poznań University of Life Sciences, Wojska Polskiego 31, 60-624 Poznań, Poland; marcellus.arnold@up.poznan.pl; 5 Department of Food Quality and Safety Management, Poznań University of Life Sciences, Wojska Polskiego 31/33, 60-624 Poznań, Poland; yolanda.rajagukguk@up.poznan.pl; 6 Department of Fishery Products, Faculty of Fisheries, Kasetsart University, Bangkok 10900, Thailand; passakorn.ki@ku.th; 7 Faculty of Agro-Industry, Chiang Mai University, Chiang Mai 50200, Thailand; supatra.ka@cmu.ac.th; 8 Faculty of Agro-Industry, Chiang Mai University, Samut Sakhon 74000, Thailand; pimonpan.k@cmu.ac.th; 9 Department of Food Science, University of Massachusetts Amherst, 102 Holdsworth Way, Amherst, MA 01002, USA; lkgrossmann@umass.edu; 10 School of Food Science and Biotechnology, Kyungpook National University, Daegu 41566, Republic of Korea; younghoonjung@knu.ac.kr; 11 Food Science and Technology Program, School of Agro-Industry, Mae Fah Luang University, Chiang Rai 57100, Thailand; saroat@mfu.ac.th; 12 Department of Food Science, Faculty of Science, Burapha University, Chonburi 20131, Thailand

**Keywords:** rheology, gelation, structure–function relationship, enzymatic modification, plant protein gels

## Abstract

The increasing demand for sustainable and functional plant-based foods has driven interest in 3D food printing technologies, which require bioinks with tailored rheological and structural properties. This study investigated the effects of transglutaminase (TGase) on the structure–function relationships of plant protein bioinks from fava bean, mung bean, pea, and soybean. TNBS assays showed a dose-dependent increase in crosslinking (27.46–64.57%), with soybean and pea proteins exhibiting the highest reactivity (*p* < 0.05). ^1^H-NMR confirmed protein-specific ε-(γ-glutamyl)lysine bond formation, and synchrotron FTIR revealed TGase-induced α-helix reduction and β-sheet enrichment, indicative of network formation across all proteins. SDS-PAGE analysis demonstrated TGase-mediated polymerization with high-molecular-weight aggregates, particularly pronounced in soybean, while SEM images revealed denser, more continuous protein networks compared to untreated samples. Rheological characterization showed enhanced viscoelasticity and shear-thinning behavior in all bioinks, supporting extrusion and post-printing stability. Textural analysis indicated improvements in hardness, springiness, cohesiveness, and chewiness across all proteins, with soybean and fava showing the most pronounced increases. These results demonstrate that TGase is a versatile tool for reinforcing plant protein networks, improving printability, structural integrity, and texture in 3D-printed foods, while highlighting protein-specific differences in response.

## 1. Introduction

Rising demand for sustainable protein sources is driving innovation in food processing, particularly in improving the texture and functionality of plant-based alternatives [1]. Among these innovations, 3D food printing has emerged as a transformative technology, offering precise control over microstructure, composition, and texture, while reducing waste and enabling personalized nutrition [2,3]. The success of 3D-printed plant-based foods depends on bio-inks with optimized rheological properties that balance printability and structural integrity. Current plant protein sources, including soy protein isolate (SPI), pea protein isolate, and wheat gluten, present significant formulation challenges in their unmodified forms [4,5,6]. In extrusion-based 3D food printing, these limitations are further amplified by processing conditions such as hydration level, shear stress during extrusion, temperature exposure during printing, and time-dependent structural recovery after deposition. Variations in these parameters can markedly influence protein unfolding, intermolecular interactions, and water distribution, thereby affecting extrusion stability and shape fidelity. These proteins exhibit suboptimal viscoelastic properties, characterized by insufficient gel strength, limited pseudoplastic behavior, and weak intermolecular network formation, which collectively compromise extrusion consistency, shape retention, and final product texture [7,8]. Successful 3D food printing requires bio-inks with shear-thinning behavior for smooth extrusion, rapid structural recovery for shape stability, and adequate mechanical strength to support multi-layer structures [5]. Furthermore, reproducibility of printed constructs is a critical consideration, as consistent dimensional fidelity and layer stacking are required for high-quality food products. Such modifications must address the fundamental molecular interactions governing protein gelation, water-binding capacity, and thermomechanical properties to achieve the precise rheological control necessary for high-quality 3D food printing.

Transglutaminase (TGase; EC 2.3.2.13) is an enzyme that catalyzes acyl-transfer reactions between γ-carboxamide groups of glutamine residues and ε-amino groups of lysine residues in proteins, forming covalent ε-(γ-glutamyl)lysine bonds [9]. This enzymatic crosslinking enhances protein network formation, improving gel strength, water-holding capacity, and structural stability in food systems [10,11]. In the context of 3D food printing, TGase has been investigated as a potential approach to modulate the structural and functional properties of plant protein-based formulations. TGase-mediated crosslinking has been reported to influence protein network formation and gelation behavior in various food systems [7,8]. Previous studies suggest that TGase treatment may influence printability and post-deposition stability of protein-based formulations [12,13]. Research has shown that TGase-modified protein matrices exhibit higher viscoelasticity and mechanical strength, essential for meat analogs that mimic the bite and texture of conventional meat [13,14,15,16]. It should be noted that the focus of this study is strictly on food-related applications, and biomedical implications are beyond the scope of this work. Thus, TGase is a promising biocatalyst for enhancing the functionality of plant-based proteins in additive manufacturing, making it an enabling technology for sustainable, high-quality, and personalized food production.

Advanced spectroscopic techniques, such as synchrotron-based Fourier transform infrared (FTIR) spectroscopy and proton nuclear magnetic resonance (^1^H-NMR), provide powerful tools to bridge this knowledge gap. Synchrotron-FTIR offers high spectral resolution for detecting protein secondary structure changes (e.g., α-helix unfolding, β-sheet formation) during TGase treatment [17,18], while ^1^H-NMR can confirm isopeptide bond formation and alterations in molecular mobility [19]. High-resolution NMR structure determination requires extracting numerous distance and orientation restraints for peptide–protein complexes [20]. These techniques can provide complementary information on structural changes in plant protein systems subjected to enzymatic treatment. Moreover, comparing multiple plant protein sources provides insight into protein-specific responses to enzymatic crosslinking, driven by differences in amino acid composition, globulin-to-albumin ratios, and tertiary structures [21]. Such comparative analysis is critical for selecting optimal proteins for precision food printing and personalized nutrition. While previous studies have demonstrated that TGase improves textural and rheological properties of plant proteins, the molecular basis of these improvements remains underexplored, especially in relation to secondary structure changes and isopeptide bond formation [13,22,23]. Furthermore, the link between molecular modifications and 3D printability metrics is not well established.

The objective of this study is to comparatively examine the effects of transglutaminase (TGase) treatment on the chemical, structural, and rheological properties of selected plant protein-based bioinks relevant to extrusion-based 3D food printing. To support this comparison, spectroscopic techniques including synchrotron-based FTIR and ^1^H-NMR were applied to qualitatively assess protein secondary structure changes and chemical environments associated with TGase treatment. A comparative framework was used to explore potential associations between TGase-induced modifications and changes in physicochemical, rheological, and printing-related properties. By bridging the knowledge gap between molecular-level structural changes and macroscopic 3D printing performance, this investigation seeks to develop evidence-based guidelines for selecting optimal plant proteins and formulation conditions for precision food printing and personalized nutrition applications.

## 2. Materials and Methods

### 2.1. Chemicals

2,4,6-Trinitrobenzenesulfonic acid solution (TNBS) was purchased from Sigma-Aldrich Chemical (St. Louis, MO, USA). Sodium phosphate dibasic heptahydrate (Na_2_HPO_4_·12H_2_O) and sodium phosphate monobasic monohydrate (NaH_2_PO_4_·H_2_O) were sourced from Fluka Chemika-BioChemika (Buchs, Switzerland). Sodium dodecyl sulphate (SDS) was obtained from Bio-Rad Laboratories (Hercules, CA, USA). Deuterium oxide (D2O) and 2,2,3,3-d4-3-(trimethylsilyl)propanoic acid sodium (TSP) were purchased from Alfa Aesar, Basel, Switzerland. TGase (ACTIVA TG-B) sourced from Ajinomoto Co., Ltd. (Tokyo, Japan) contained 0.5% TGase (50 U/g activity), 2.5% sodium carbonate, 2.0% silicon dioxide, and 95% milk protein as carrier. Mung bean protein isolate (MP) (85% protein), pea protein isolate (PP) (85% protein), fava bean protein isolate (FP) (85% protein), and soybean protein isolate (SP) (90% protein) were purchased from Chanjao Longevity Co., Ltd. (Bangkok, Thailand).

### 2.2. Preparation of TGase-Crosslinked Protein Samples

Plant protein isolates (FP, MP, PP, and SP) were dispersed in phosphate buffer (pH 7.0) to a final protein concentration of 1% (*w*/*v*). This low-concentration system was selected to allow accurate structural characterization (TNBS and ^1^H-NMR), which cannot be reliably performed in high-viscosity bioink matrices. TGase activity was expressed and controlled on a protein-mass basis (U/g plant protein) throughout the study to allow direct comparison among different plant protein isolates. TGase was prepared at 0.5 U/mL in the same buffer and mixed with the protein dispersions at enzyme-to-substrate ratios corresponding to 10, 20, and 50 U/g plant-based protein, based on reported conditions effective for plant-protein crosslinking without inducing excessive gelation. For crosslinking, 1000 µL of protein solution was combined with 1000 µL of TGase solution and incubated at 55 °C for 2 h in water bath (°LAUDA, H 22, Lauda-Königshofen, Germany). The reaction was terminated by heating at 95 °C for 10 min to inactivate TGase; the same heat treatment was applied to non-TGase controls to ensure valid comparisons. Samples were cooled, freeze-dried, and stored for subsequent synchrotron-FTIR, TNBS, and ^1^H-NMR analyses. Crosslinked samples were denoted by adding the letter “C” to each protein abbreviation (e.g., FP-C, MP-C, PP-C, SP-C).

### 2.3. Determination of Accessible Primary Amines by TNBS Assay

The TNBS assay was used to quantify accessible primary amine groups in protein samples, following the procedure described by Sai-Ut et al. [24], with modifications to accommodate plant-based protein samples. Briefly, 100 μL of each sample was mixed with 1.0 mL of 0.20 M phosphate buffer (pH 8.2) and 0.8 mL of 0.01% (*v*/*v*) TNBS solution. The mixture was incubated in the dark at 50 °C for 20 min to allow derivatization of free α-amino groups. The reaction was terminated by adding 1.0 mL of 0.1 M sodium sulfite, followed by cooling at room temperature for 5 min. Absorbance was measured at 420 nm using a UV–Vis spectrophotometer. To quantify the extent of TGase-mediated reduction in accessible primary amine groups, the amount of free α-amino groups remaining after enzymatic treatment was compared to a control sample prepared under identical conditions but without active TGase (heat-inactivated enzyme). The degree of crosslinking was calculated based on the reduction in TNBS-reactive primary amines, expressed in L-leucine equivalents.

### 2.4. H-NMR-Based Analysis

For the ^1^H-NMR analysis, sample preparation, spectral acquisition, and metabolite identification were performed following the procedures described by Sai-Ut et al. [25], with minor modifications to suit plant protein matrices. Briefly, 100 mg of the treated protein sample was dissolved in 2000 µL of 80% (*v*/*v*) deuterium oxide containing 80 mM phosphate buffer at pH 7.4, supplemented with 0.18 mM 3-(trimethylsilyl)propionic-2,2,3,3-d_4_ acid sodium salt (TSP) as the internal standard. The mixture was centrifuged at 8500 rpm for 5 min. The 600 µL of supernatant was collected with a pipette and placed in 5 mm NMR tube. All ^1^H-qNMR experiments were performed using a Bruker Avance III HD 400 MHz NMR spectrometer, equipped with a 5 mm CryoProbe prodigy (Double Resonance Broadband Observe with 19F probe) at 25 °C. The ^1^H-qNMR spectra of samples were collected using the following parameters: pulse program zg; relaxation delay 70 s; pulse width 8.89 µs; number of scan 16 and 69 K data; sweep width 24 ppm and center of spectrum is 9.00 ppm. Line broadening (lb = 03 Hz) was used while processing. The internal standard shift establishes the reference point for all chemical shifts of metabolites, facilitating their identification. The intensities of the ^1^H-NMR spectra were normalized to the total intensity and segmented into integrated regions of equal width within the chemical shift range. Compound identification was achieved using information from 1D and 2D NMR experiments, statistical total correlation spectroscopy (STOCSY), and database searches in the Biological Magnetic Resonance Data Bank (BMRB) and the Birmingham Metabolite Library (BML).

### 2.5. Synchrotron Radiation-Based FTIR

The structural characteristics of crosslinked protein powders were examined using synchrotron-FTIR spectroscopy. A Bruker Tensor 27 infrared spectrometer, equipped with deuterated alanine-doped triglycine sulfate (DTGS) detectors, was employed following the protocol outlined by Pongsetkul et al. [26]. Each treatment group underwent six replications. SR-FTIR spectral data were obtained using a Vertex 70 spectrometer paired with a Hyperion 2000 IR microscope, controlled via OPUS 7.5 software (Bruker Optics, Ettlingen, Germany). Data acquisition ranged from 4000 to 400 cm^−1^ with a spectral resolution of 4 cm^−1^, using 64 scans for each of the 30 replicates, resulting in 180 spectra per treatment (30 spectra × 6 replicates). Spectral processing was performed using Unscrambler X (version 10.5, Camo Analytics, Oslo, Norway), applying the Savitzky–Golay algorithm with a third-order polynomial and 13 smoothing points to compute the second derivative, thus minimizing baseline variations. Normalized spectra were examined within the regions of 3000–2800 cm^−1^ and 1800–900 cm^−1^, enabling enhanced resolution of overlapping peaks. Principal component analysis (PCA) was conducted on mean-centered second-derivative spectra to evaluate treatment-related spectral variation, following established FTIR chemometric practices for protein systems [27] to assess variations and reduce data dimensionality. The relative intensity of specific absorption bands was quantified as the percentage of the integrated area, targeting regions such as 3000–2800 cm^−1^ (C–H stretching from lipids), 1740 cm^−1^ (C=O ester from lipids), 1700–1600 cm^−1^ (amide I), 1600–1500 cm^−1^ (amide II), 1338 cm^−1^ (amide III), and 1250–900 cm^−1^ (associated with carbohydrates and glycogen). Additionally, the proportions of protein secondary structure elements α-helix, β-sheet, β-turn, and antiparallel configurations, were calculated using Gaussian and Lorentzian curve fitting functions within OPUS 7.8 software (Bruker Optics, Ettlingen, Germany). After integrating subpeak areas within this range, results were expressed as relative percentages.

### 2.6. Rheology Properties Analysis

Plant protein powders were used to achieve a final concentration of 20% plant-based protein in the bioink. The powders were mixed with 1.0% and 5.0% TGase powder, corresponding to low (2.5 U/g plant-based protein) and high (12.5 U/g plant-based protein) enzyme concentrations, respectively. Distilled water was then added to bring the total formulation to 100%. The mixtures were gently stirred at 25 °C for 5 min before measurement. Rheological measurements were performed using an Anton Paar MCR 302 rheometer (Anton Paar GmbH, Graz, Austria) equipped with parallel plate geometry (25 mm diameter) at a 1 mm gap, following the method of Rahman et al. [28]. To determine the linear viscoelastic region (LVR), amplitude sweep tests were first conducted at 25 °C over a strain range of 0.01–100% at a constant frequency of 1 Hz. Based on these results, a strain of 0.1%, which lay well within the LVR for all samples, was selected for subsequent tests. Frequency sweep tests were conducted at 0.1% strain within the linear viscoelastic region, with frequency ranging from 0.1 to 100 Hz at 25 °C. Storage modulus (G′), loss modulus (G″), and loss tangent (tan δ) were recorded as functions of frequency. Temperature sweep tests were performed at 0.1% strain and 1 Hz frequency. Samples were heated from 10 to 90 °C at 1 °C/min, then cooled to 10 °C at the same rate. Changes in G′ during heating and cooling cycles were monitored to evaluate thermal gelation behavior and structural recovery.

### 2.7. Electrophoretic Analysis

Protein patterns of crosslinked samples were analyzed using SDS-PAGE [29]. Samples (1.0 g) were dissolved in 9 mL of 5% (*w*/*v*) SDS, heated at 85 °C for 1 h, and the supernatant was mixed (1:1, *v*/*v*) with sample buffer (0.5 M Tris-HCl, pH 6.8, containing 4% SDS, 20% glycerol, and 10% β-mercaptoethanol), then boiled for 3 min. Protein (15 μg) was loaded onto a 7.5% resolving gel with a 4% stacking gel and electrophoresed at 15 mA per gel using a Mini-PROTEAN^®^ Tetra Cell (Bio-Rad, Hercules, CA, USA). Gels were stained with 0.1% (*w*/*v*) Coomassie Blue R-250 in 15% methanol and 5% acetic acid, then destained with 30% methanol and 10% acetic acid.

### 2.8. Plant-Based Protein Bioinks Preparation and 3D Printing Process

Plant-based protein bioinks were formulated by combining 20% plant-based protein content, 1% (*w*/*w*) xanthan gum, 1% (*w*/*w*) paprika oleoresin, and distilled water (balance to 95–99%). All components were homogenized using a handheld mixer (Elon Gen 2, Spring Green Evolution, Bangkok, Thailand) for 5 min. The formulations were sealed in polyethylene film and stored at 4 °C for 12 h to ensure complete hydration and uniform water distribution, which is critical for reproducible extrusion and enzymatic activity. After hydration, TGase was incorporated at 1.0–5.0% (*w*/*w*), corresponding to low and high enzyme activity levels selected based on preliminary screening and literature reports to induce sufficient crosslinking without premature gelation. The mixtures were homogenized for an additional 2 min and immediately transferred into 50 mL syringes for printing.

Three-dimensional printing was conducted using an extrusion-based FoodBot 3D Printer (Changxing Shiyin Technology, Hangzhou, China) under controlled ambient conditions (25 ± 2 °C, 45 ± 5% relative humidity). Printing parameters were selected based on preliminary optimization trials to balance extrusion continuity, layer adhesion, and shape fidelity across all formulations. The optimized parameters were: nozzle diameter of 1.2 mm, printing and retraction speeds of 20 mm/s, layer height of 1.2 mm, and infill density of 90%. These settings ensured stable filament deposition and minimized structural collapse during multi-layer printing. Three standardized geometries (cube, cylinder, and heart; 20 × 20 × 6 mm) were fabricated from CAD models to assess printability and dimensional stability. Following printing, samples were incubated at 55 °C for 2 h to promote TGase-mediated crosslinking, allowing covalent network formation to stabilize the printed structures. Dimensional accuracy was evaluated immediately after printing and after incubation using digital calipers (±0.01 mm precision; Mitutoyo, Kawasaki, Japan) by comparing printed dimensions with the original CAD designs. Printing accuracy for each dimension was calculated according to Equation (1),

(1) $$\mathrm{Printing}\;\mathrm{accuracy}\;(\%)=\left(1-\frac{\left|Measured\;dimension-CAD\;dimension\right|}{CAD\;dimension}\right)\times 100$$

where the “measured dimension” corresponds to the experimental value and the “CAD dimension” refers to the intended value from the 3D model.

### 2.9. Texture Profile Analysis (TPA)

Textural properties of protein pastes and 3D-printed gels were evaluated using TPA under raw-set and thermally treated conditions. Cylindrical samples (25 mm diameter × 20 mm height) were equilibrated to 25 ± 2 °C prior to measurement. Texture analysis was performed using a TA.XTplus texture analyzer (Stable Micro Systems, Godalming, UK) with a 35 mm cylindrical probe (P/35) and 1.0 kg load cell. Raw-set samples were analyzed immediately, while thermally treated gels were incubated at 55 °C for 2 h to promote TGase crosslinking, then cooled to 25 ± 2 °C before testing. The TPA protocol included a trigger force of 5 g, pre-test, test, and post-test speeds of 2 mm/s, 50% compression of sample height, and a 5 s interval between cycles. Textural parameters were calculated from force–time curves. For protein pastes (after printing), hardness and adhesiveness were measured; for 3D-printed gels (after incubation), six parameters were assessed: hardness, adhesiveness, springiness, cohesiveness, gumminess, and chewiness.

### 2.10. Scanning Electron Microscopy (SEM)

Microstructural analysis of TGase-treated plant-based protein gels was performed following the method described in Section 2.8. Gel samples were cut into (5 × 5 × 2 mm) pieces and fixed in 2.5% glutaraldehyde (0.1 M phosphate buffer, pH 7.2) at 4 °C for 12 h, then washed three times with phosphate buffer (20 min each). Samples underwent graded ethanol dehydration (50%, 80%, 90%, 99.5%; 20 min each) followed by critical point drying. Dried specimens were fractured to expose internal structure, mounted on aluminum stubs, and sputter-coated with gold (~10 nm). Imaging was conducted using a SEM (LEO 1450VP, Oberkochen, Germany) at 10–15 kV acceleration voltage and 50–100× magnification to evaluate network density, porosity, and aggregation patterns.

### 2.11. Statistical Analysis

All experiments were conducted at least in triplicate. Data were analyzed by one-way ANOVA to assess variance, and mean comparisons were performed using Tukey’s HSD test at a 95% confidence level (*p* < 0.05).

## 3. Results and Discussion

### 3.1. Impact of Tgase on Protein Crosslinking in Different Plant-Based Protein

#### 3.1.1. Accessible Primary Amines by TNBS

The TNBS assay results (Table 1) demonstrated a clear TGase concentration-dependent decrease in TNBS-reactive primary amines for all four plant protein isolates, indicating progressive enzymatic modification associated with protein network formation. Both SP and PP showed the highest reduction in accessible primary amines at 50 U/g plant-based protein of TGase (64.56 ± 1.55% and 62.34 ± 3.60%, respectively; *p* > 0.05), indicating that they were similarly effective TGase substrates. Their strong reactivity likely reflects a combination of abundant glutamine and lysine residues and favorable structural accessibility that facilitates ε-(γ-glutamyl)lysine bond formation [30]. Although the formulation contains a small proportion of casein to aid dispersion and stabilize the system, its high TGase reactivity requires consideration when interpreting crosslinking results. Soybean’s 7S and 11S globulins and pea’s vicilin- and legumin-rich fractions both provide flexible, partially denaturation-prone conformations that enhance enzyme accessibility [31]. In addition, soy protein isolates are well documented to contain relatively high levels of both glutamine and lysine residues, largely contributed by the major storage proteins glycinin (11S) and β-conglycinin (7S), which together provide a high density of reactive amide and ε-amino groups accessible for enzymatic crosslinking. Similarly, pea protein isolates, dominated by vicilin and legumin fractions, have been reported to exhibit moderate-to-high lysine contents and sufficient glutamine availability, supporting effective TGase reactivity under suitable hydration and processing conditions [31,32,33,34]. MP exhibited moderate crosslinking (56.26 ± 5.22% at 50 U/g plant-based protein), which may stem from its more compact tertiary structure that limits surface exposure of reactive residues [35]. FP displayed the lowest reduction in accessible primary amines at 10 U/g plant-based protein of TGase (27.46 ± 3.57%) but approached mung levels at higher enzyme doses, suggesting partial structural flexibility that becomes accessible upon increased TGase activity [36,37]. These differences in the reduction of accessible primary amines, reflecting relative TGase reactivity, have direct implications for 3D food printing performance, as proteins exhibiting higher TGase responsiveness tend to form stronger and more cohesive networks, thereby improving gel strength, printability, and dimensional stability of plant-based bioinks. Although the formulation contains a small proportion of casein to aid dispersion and stabilize the system, its high TGase reactivity requires consideration when interpreting crosslinking results. However, plant proteins remain the dominant contributors to TGase responsiveness, as evidenced by (i) distinct crosslinking differences among FP, MP, PP, and SP despite identical casein content, and (ii) SDS-PAGE polymerization patterns aligned with plant protein structural traits rather than uniform casein-driven behavior. These findings indicate that while casein acts as a minor co-gelling component, TGase responses are primarily governed by the plant protein matrices.

#### 3.1.2. Analysis of Protein Crosslinking by NMR

**Figure 1:**
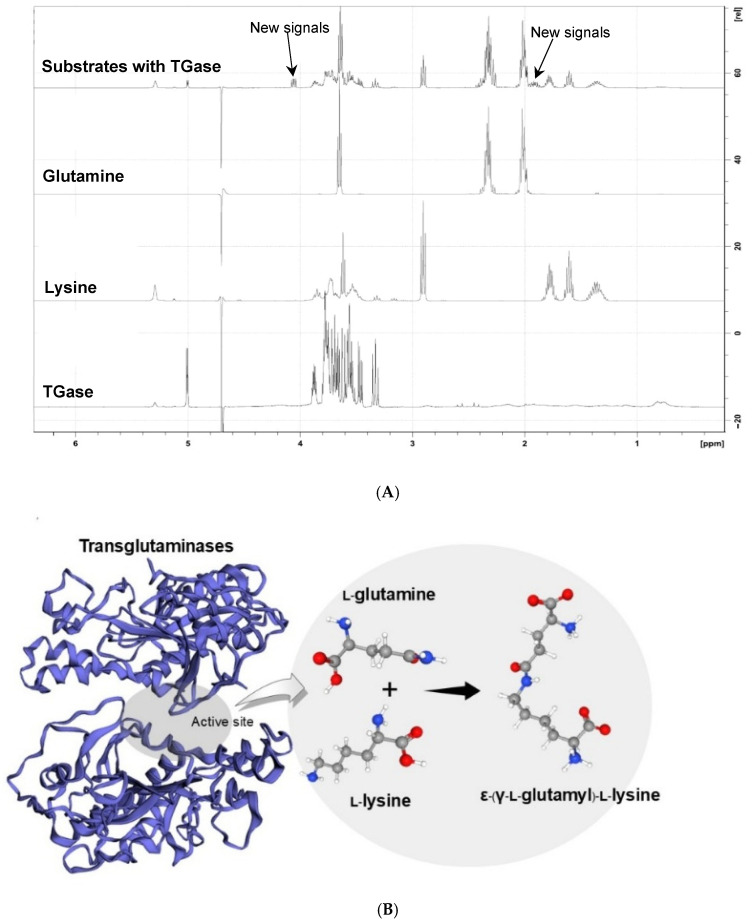
^1^H-NMR spectra of the single model substrate (glutamine, lysine) in the absence and presence of TGase (**A**) and The TGase structure and formation of crosslinking generated by EzMol modelling software (http://www.sbg.bio.ic.ac.uk/ezmol/) (PDB: 1IU4) (**B**).

The ^1^H-NMR spectra of glutamine, lysine, and TGase mixtures (Figure 1A) revealed the emergence of signals at δ 1.9 and 4.1 ppm, absent in the individual substrates or enzyme alone. The δ 1.9 ppm signal is attributed to lysine ε-methylene protons involved in ε-(γ-glutamyl)lysine crosslinks, while δ 4.1 ppm corresponds to α-protons of glutamine residues engaged in the same reaction, consistent with prior MTGase studies [19,38]. In the present study, these resonances are used solely as qualitative indicators of TGase-mediated modification rather than as quantitative measures of crosslinking extent, as calibration, linearity, and absolute quantification were not established. The selected enzyme-to-substrate ratios and subsequent heat inactivation were applied to promote enzymatic modification while terminating TGase activity prior to downstream analyses, thereby ensuring consistent reaction conditions across samples and minimizing uncontrolled post-reaction changes.

**Figure 2:**
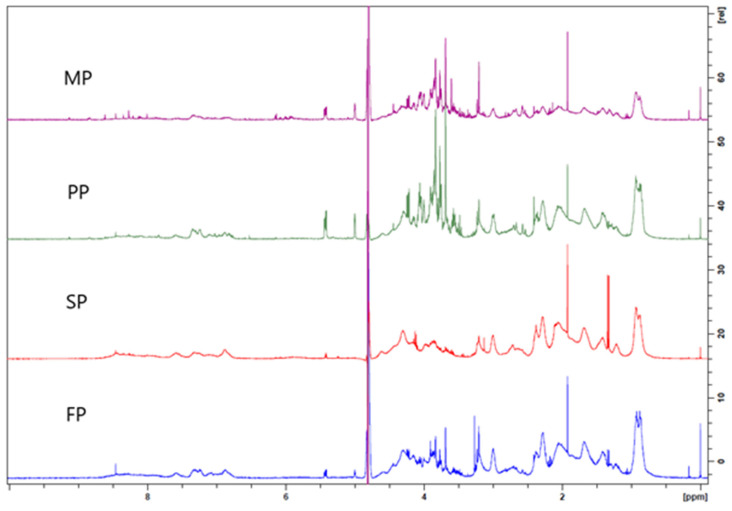
^1^H-NMR spectra of plant-based protein isolates after treated with TGase.

^1^H-NMR spectra of plant protein mixtures with varying TGase concentrations (Figure 2) showed protein-specific spectral patterns reflecting amino acid composition and structural features. The spectra represent a superposition of random coil signals from constituent residues [39]. Rather than enabling direct quantification of crosslink density, the NMR analysis provides comparative insight into relative molecular mobility and structural complexity among protein systems under identical treatment conditions. Several low-molecular-weight compounds (e.g., valine, alanine, methionine, lactate, succinate, choline, betaine, glucose, inosine, AMP, nicotinate, and phenolic derivatives; Supplementary Table S1) were detected. These signals reflect soluble components present in hydrated protein preparations and are not interpreted as direct evidence of protein–protein binding or enzymatic crosslink formation [40,41,42]. Notably, SP spectra exhibited broader peaks relative to MP, PP, and FP, indicative of larger macromolecular assemblies and reduced mobility due to more extensive TGase-mediated crosslinking. Sharper peaks in FP, MP, and PP suggest comparatively limited crosslinking. Signal overlap in the 1.9–4.1 ppm region and other congested areas reflect the inherent complexity of plant proteins, including high molecular weight and diverse amino acid compositions, which challenge precise resonance assignment and quantification [43,44]. Deuterium labeling or spectral deconvolution may improve resolution for more accurate assessment [45]. Therefore, ^1^H-NMR serves as a complementary qualitative technique to support evidence of TGase-induced molecular modification, while acknowledging that direct quantification of crosslinking requires additional targeted analytical methods.

#### 3.1.3. Analysis of Protein Crosslinking by SR-FTIR

**Figure 3:**
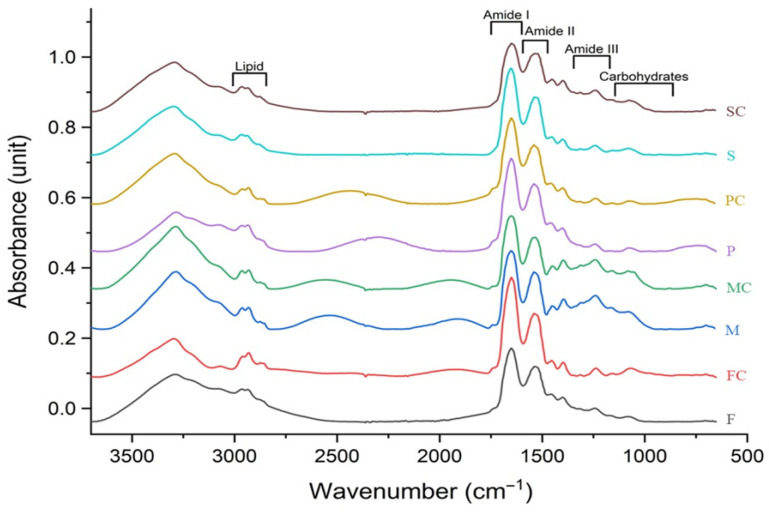
Average SR-FTIR spectra (4000–400 cm^−1^) of plant proteins with and without TGase (10 U/g protein).

Figure 3 shows that the dominant vibrational regions across all samples are Amide I (~1600–1700 cm^−1^) and Amide II (~1500–1600 cm^−1^), collectively accounting for approximately 80% of the total integrated area. These regions correspond to the main functional groups of the protein backbone, specifically C=O stretching (Amide I) and N–H bending/C–N stretching (Amide II), both of which are sensitive to protein secondary structure [46]. Amide I contributed most prominently in all samples, indicating its high responsiveness to structural modifications and making it a reliable marker for evaluating TGase-induced conformational changes. TGase treatment produced protein-specific alterations in the Amide I and II regions. FP-C and MP-C exhibited increased Amide I area, consistent with enhanced carbonyl stretching due to crosslink formation. These covalent bonds likely increase the density of ordered structures or exposure of backbone carbonyl groups, amplifying the Amide I signal [47,48]. Conversely, PP-C and SP-C showed a decrease in Amide I area following TGase treatment, suggesting structural compaction or intramolecular crosslinking that masks carbonyl groups and reduces IR absorbance. For the Amide II region, FP-C, MP-C, and SP-C demonstrated increased intensity, indicating enhanced N–H bending and C–N stretching associated with hydrogen bonding and network formation [49]. PP-C, however, displayed a reduced Amide II signal, implying distinct conformational adjustments or partial unfolding that limit N–H/C–N accessibility, consistent with its comparatively lower susceptibility to TGase-mediated crosslinking.

Table 2 and Figure S1 and present deconvolution of Amide I bands, revealing systematic changes in secondary structure following TGase treatment. All proteins exhibited reduced α-helix content after TGase addition, consistent with enzymatic crosslinking disrupting helical stability and promoting conversion into more thermodynamically stable β-sheet structures [23,50]. Similar results were reported by San et al. [51], who observed that TGase cross-linking led to a reduction in α-helix content and an increase in β-sheet content in black bean protein. Furthermore, Peng et al. [34] reported increased β-sheet content with concomitant β-turn reduction following TGase treatment, correlating positively with enzyme concentration. The consistent increase in antiparallel β-sheet content suggests TGase promotes ordered inter-chain arrangements through intermolecular crosslinks, facilitating extended protein aggregation [16]. However, the functional impact of these structural changes is protein-dependent, as differences in amino acid composition, globulin-to-albumin ratio, and tertiary structure influence how β-sheet formation translates into macroscopic properties. Importantly, PP displayed the largest TGase-induced increase in β-sheet content and the greatest reduction in α-helix among all samples, indicating the most pronounced secondary-structure rearrangement. This discrepancy highlights that β-sheet enrichment alone does not fully determine gel strength or printability; network formation also depends on overall protein accessibility and interactions with other formulation components. This contrasts with the crosslinking and rheological results, where both PP and SP showed similarly high TGase responsiveness. The consistent increase in antiparallel β-sheet structures across proteins suggests that TGase promotes more ordered intermolecular arrangements, although PP demonstrated the strongest shift toward these extended configurations, while MP exhibited comparatively limited antiparallel β-sheet development [16]. These β-sheet-enriched structures are characteristic of enzyme-induced protein networks and contribute to improved gelation and structural integrity [18]. Accordingly, proteins showing more extensive β-sheet formation (particularly PP, and to a slightly lesser extent SP) are likely to form cohesive, stable networks after extrusion, enhancing rheological behavior, texture, and shape retention in 3D-printed constructs, but the effect may vary depending on protein type and formulation context.

**Figure 4:**
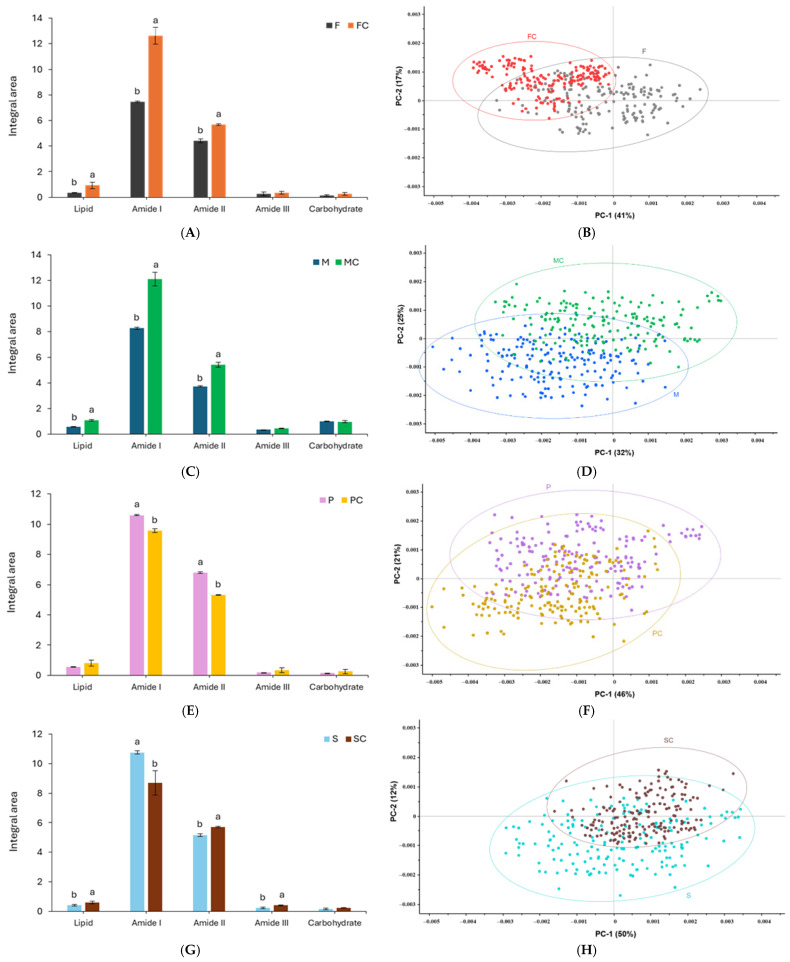
Integral area (%) of biomolecules (4000–400 cm^−1^) (**left**) and PCA score plot (**right**) of plant proteins with and without T-Gase (10 U/g protein). FP: fava bean protein isolate (**A**,**B**); MP: mung bean protein isolate (**C**,**D**); PP: pea protein isolate (**E**,**F**); SP: soy bean protein isolate (**G**,**H**); the suffix ‘C’ denotes TGase-crosslinked samples. Different lowercase letters indicate significant differences between samples (*p* < 0.05).

PCA of the FTIR spectra (Figure 4) effectively discriminated untreated and TGase-treated samples across all protein systems, with the clearest separation occurring along PC1. For FP, PC1 accounted for 41% of the variance and distinctly separated TGase-treated samples (Figure 3). The corresponding loading plots indicated that discrimination was driven by changes in lipid-associated CH stretching at 2964.23 cm^−1^ and antiparallel β-sheet contributions near 1680.94 cm^−1^ for FP-C, whereas untreated FP showed dominant α-helix bands at 1646 and 1658.9 cm^−1^ (Supplementary Figures S2 and S3). These band assignments are consistent with established FTIR literature describing Amide I sub-band ranges for α-helix (≈1645–1660 cm^−1^) and β-sheet components (≈1610–1640 and 1670–1695 cm^−1^) [52,53]. For MP-C and SP-C, the major discriminative loadings occurred within antiparallel and β-sheet regions, whereas PP-C exhibited strong β-turn and β-sheet contributions following TGase treatment. In SP-C, peaks at 2967.02 cm^−1^ and 1237–1239 cm^−1^ (Amide III) suggest alterations in side-chain mobility and backbone rearrangements, consistent with established Amide III assignments linked to C–N stretching and N–H bending [54]. SP displayed strong positive PC1 loadings (50%) at 1682.47 and 1693.91 cm^−1^ (antiparallel β-sheet) and 1237.79–1239.98 cm^−1^ (Amide III), indicating β-sheet stabilization after TGase treatment (Supplementary Figure S3). Conversely, negative PC1 loadings at 1629.17 and 1638.39 cm^−1^ reflected native or less ordered β-sheet structures that diminished upon crosslinking. The presence of 2967.02 cm^−1^ in the loading profiles aligns with lipid-associated CH vibrations and hydrophobic interactions that can shift as protein networks reorganize.

Together, these findings indicate that TGase influences both lipid–protein interactions and secondary-structure elements, reflected by shifts within the Amide I and III regions. The combined SR-FTIR, PCA, and secondary-structure fitting analyses confirm that TGase induces protein-dependent conformational rearrangements, increasing ordered β-sheet and antiparallel structures, in agreement with previous reports of TGase-mediated structural stabilization in plant proteins [55]. These structural modifications have functional implications, particularly for gelation behavior and 3D printability.

#### 3.1.4. Protein Pattern by SDS-PAGE

**Figure 5:**
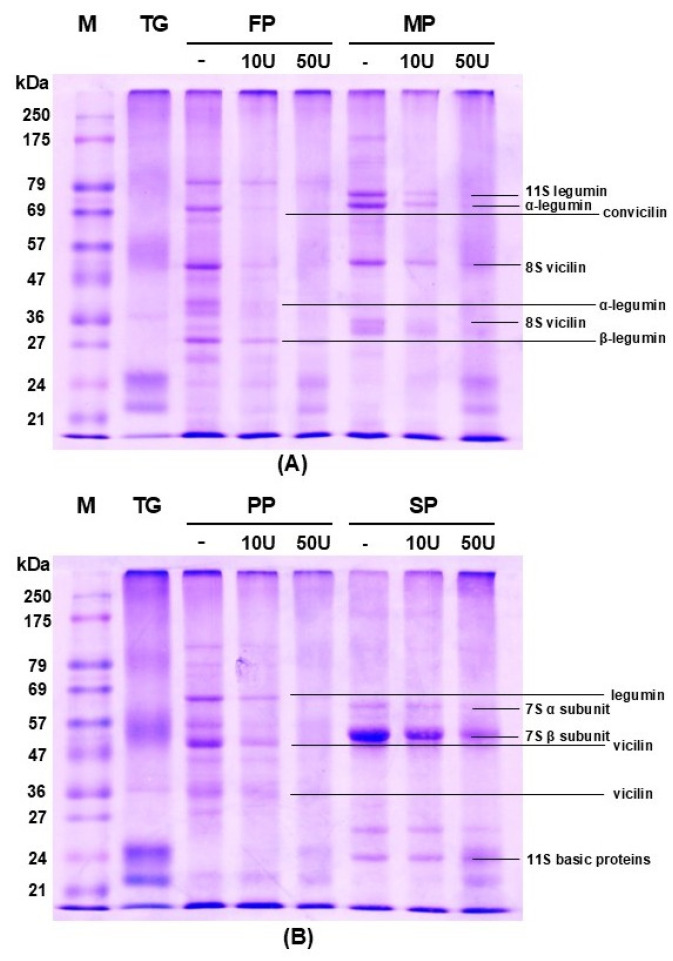
SDS-PAGE profile of various plant proteins treated with TGase at different concentrations. M: molecular weight marker (21–250 kDa); TG: Transglutaminase; FP: fava bean protein isolate; MP: mung bean protein isolate (**A**); PP: pea protein isolate; SP: soy bean protein isolate (**B**); Lanes 3–8: plant protein samples treated with TGase at varying concentrations. Specifically, Lane 3 and 6: untreated control (-); Lanes 4 and 7: protein treated with TGase of 10 U/g plant-based protein (10 U); Lanes 5 and 8: protein treated with TGase of 50 U/g plant-based protein (50 U).

SDS-PAGE analysis effectively demonstrated the differential crosslinking behavior of plant proteins treated with TGase, with distinct banding patterns observed (Figure 5). The extent of crosslinking varied with TGase concentration (10 U/g and 50 U/g protein), highlighting both enzyme dose-dependency and differences in protein structural susceptibility. In untreated samples, characteristic bands corresponded to storage globulin subunits. SP exhibited dominant bands at ~55 and 62 kDa, representing the α and β subunits of β-conglycinin (7S), and bands at ~26 and 24 kDa corresponding to glycinin (11S) subunits [31]. FP showed bands at ~80, 69, 52, and 28 kDa, associated with vicilin and legumin subunits [56]. M displayed bands at ~77, 72, and 50 kDa, likely related to vicilin-like 7S globulins [57]. PP presented bands at ~66 and 50 kDa, corresponding to legumin and vicilin, respectively [58]. Upon TGase treatment, the intensity of these bands progressively decreased, with the emergence of high molecular weight (HMW) aggregates accumulating at the gel top or stacking region. This reflects TGase-catalyzed formation of ε-(γ-glutamyl)lysine isopeptide bonds, linking glutamine and lysine residues within and between protein molecules. Increasing enzyme concentration enhanced this effect, supporting the dose-dependent nature of crosslinking [34]. Among the proteins tested, SP exhibited the most extensive polymerization, as evidenced by smeared, retained bands at the top of the gel. In contrast, MP showed limited crosslinking, retaining visible monomeric bands and producing fewer HMW aggregates. This may result from limited accessibility or availability of reactive residues or structural conformations that hinder enzyme interaction, consistent with known TGase substrate specificity [59]. The reduction in low-molecular-weight bands and the corresponding accumulation of HMW species provide molecular evidence of TGase-induced crosslinking. These structural modifications have functional implications, enhancing properties such as gelation, viscoelasticity, and mechanical strength. Such improvements are particularly relevant for plant-based food applications, including meat analogues and 3D food printing, where protein network formation is critical for texture and stability.

### 3.2. Rheological Analysis

#### 3.2.1. Viscoelastic Properties

**Figure 6:**
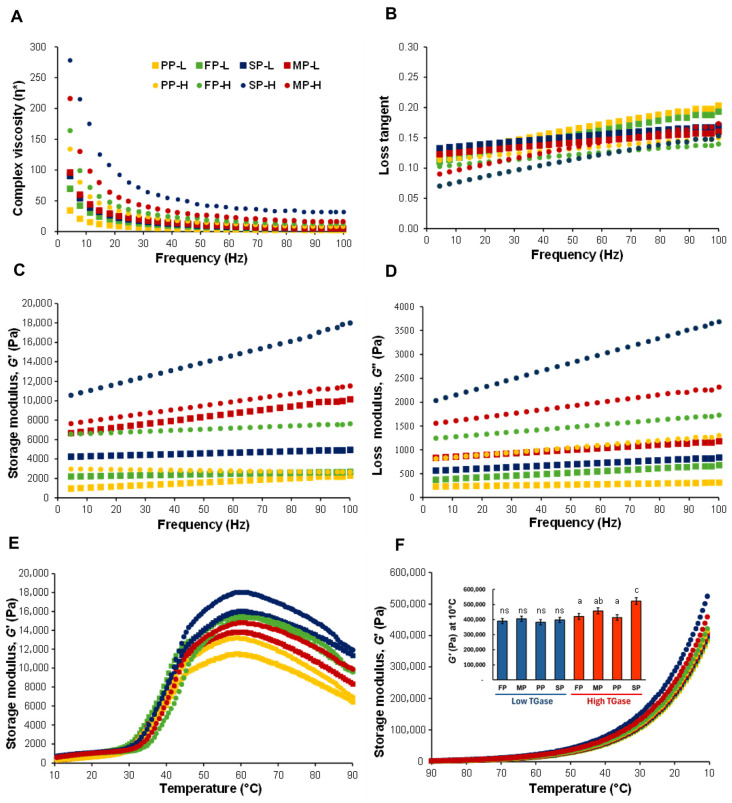
Rheological behavior of plant protein bioinks with low (6.25 U/g plant-based protein, suffix ‘L’ (square symbol)) and high (12.5 U/g plant-based protein, suffix ‘H’ (circle symbol)) TGase: Complex viscosity (η*) (**A**), loss tangent (tan δ) (**B**), storage modulus G′ (**C**), loss modulus G″ (**D**), and temperature-dependent G′ during heating (10–90 °C, (**E**)) and cooling (90–10 °C, (**F**)). Colors and symbols indicate plant protein type: PP (yellow), FP (green), SP (blue), and MP (red). Different lowercase letters indicate significant differences between samples (*p* < 0.05), ns = not significant.

The viscoelastic behavior of the plant protein-based bioinks was strongly affected by TGase treatment (Figure 6). All formulations demonstrated clear shear-thinning behavior, which is essential for extrusion-based 3D printing. At the applied TGase level of 6.25 U/g plant-based protein (2.5% TGase powder), the bioinks consistently exhibited enhanced pseudoplasticity, promoting smooth flow through the nozzle while maintaining sufficient structure after deposition. Among the proteins tested, SP showed a substantial absolute reduction in dynamic viscosity following TGase treatment (from ~270 Pa to ~50 Pa; Δ ≈ 220 Pa), supporting its rapid structural breakdown under increasing frequency. However, when considering relative changes, MP (~7-fold), PP (~6.5-fold), and FP (~5.3-fold) proteins experienced proportionally greater viscosity reductions than SP (~5.4-fold), indicating that TGase consistently weakened network resistance across all protein matrices. This combined absolute–relative perspective emphasizes that while SP exhibited the highest initial viscosity and largest absolute decrease, the enzyme-induced softening effect was broadly comparable among the four proteins. To evaluate shear-thinning behavior and predict printability, oscillatory frequency sweep tests were conducted, as they provide insights into the viscoelastic response and structural recovery of the protein network under controlled small deformations. While these oscillatory tests provide indirect indications of flowability and gel strength relevant to printing, they do not replace direct extrusion measurements, and shear recovery or thixotropic behavior were not assessed in this study. Observed extrusion performance during 3D printing further confirms that these bioinks exhibit appropriate shear-thinning behavior for smooth deposition and dimensional stability. In agreement with these results, Cheng et al. [13] demonstrated that TGase-treated soy protein based ink exhibited shear-thinning behavior with decreasing apparent viscosity at higher shear rates, and noted that TGase-treated groups maintained higher apparent viscosity compared to non-enzymatic controls. TGase crosslinking improved structural recovery and network formation post-deposition, crucial for maintaining printed geometry. Dynamic viscoelastic behavior, assessed through loss tangent (tan δ = G″/G′), revealed frequency-dependent variations within a predominantly solid-like (gel) regime (Figure 6B). SP showed comparable tan δ values at low frequencies regardless of TGase concentration, but high-TGase samples exhibited steeper increases at higher frequencies, suggesting reduced structural resilience despite enhanced crosslinking. Storage (G′) and loss (G″) moduli increased with oscillatory frequency (1–100 Hz), characteristic of weak gel systems (Figure 6C,D). Low TGase concentrations consistently yielded higher moduli, suggesting optimal network integrity without excessive rigidity. Both G′ and G″ increased with oscillatory frequency due to enhanced internal friction, with G′ > G″ indicating predominantly elastic behavior of the SP-based ink [4]. In addition, variations in viscoelastic properties correlated with structural modifications observed in NMR and SR-FTIR analyses. TGase-mediated α-helix to β-sheet transitions and increased antiparallel β-sheet content enhanced protein network cohesion, which is reflected in higher G′ and G″ values and improved shape fidelity. Among the protein sources, SP exhibited the highest G′ and G″, followed by MP, whereas FP and PP showed the lowest values. These differences may reflect variations in globulin composition and protein–protein interactions, as reported in previous studies on legume proteins [56,60,61]. In all samples, the predominance of G′ over G″ confirmed gel-like behavior with elastic properties, which is critical for extrusion-based 3D printing applications that require shape fidelity and structural stability. Excessive TGase loading (>5% powder) was found to cause nozzle clogging due to accelerated crosslinking, but such conditions were not used in further study.

#### 3.2.2. Temperature Sweep Measurements

The evolution of storage modulus (G′) during heating (10–90 °C) provides critical insights into thermal gelation, which directly affects the processing stability of plant protein-based bioinks (Figure 6E). All samples exhibited a marked increase in G′ beginning at approximately 40 °C, reaching peak values around 60 °C. This behavior reflects protein unfolding and subsequent aggregation, leading to the formation of a 3D network [62]. Among the proteins tested, SP displayed the highest G′ values within the 40–60 °C range, indicating superior gel-forming ability compared to other plant proteins. Such thermal responsiveness is advantageous for 3D printing since it promotes structural integrity during post-extrusion setting. Above 60 °C, a decline in G′ was observed, likely due to excessive denaturation and partial network disassembly, which reduces gel rigidity [63]. The rheological properties of inks are strongly dependent on heating time, with Yu et al. [64] showing that extended preheating increased G′ values and promoted sol–gel transition. Printing temperature critically affects final output quality by causing protein denaturation that reveals hydrophobic sites available for covalent cross-linking [65]. During cooling (90–10 °C), G′ progressively increased in all samples, with significant enhancement below 60 °C, indicating cold-set gelation facilitated by TGase-mediated crosslinks and intermolecular aggregation (Figure 6F). SP exhibited the highest cold-set G′ values, followed by MP, suggesting stronger network formation and improved dimensional stability at ambient conditions. All formulations displayed shear-thinning behavior, a rheological characteristic essential for smooth extrusion through printer nozzles. Frequency sweep measurements confirmed that all bioinks displayed solid-like, elastic-dominated behavior (G′ > G″, tan δ < 1) across the 1–100 Hz range, supporting layer stacking during 3D printing. Tan δ values decreased with increasing TGase concentration, highlighting enhanced elasticity and network cohesion. While SP showed minimal frequency dependence at low TGase levels (1–10 Hz), higher TGase concentrations resulted in slight increases in tan δ at higher frequencies (50–100 Hz), consistent with reduced structural resilience under dynamic shear. MP, PP, and FP exhibited proportionally similar trends, indicating that TGase-induced crosslinking consistently strengthens elastic behavior while maintaining shear-thinning flow for extrusion. Thus, these data distinguish the respective contributions of thermal aggregation and enzymatic crosslinking: heating primarily drives partial unfolding and aggregation, whereas TGase-mediated covalent crosslinks enhance structural stability during both heating and cooling, improving printability, shape fidelity, and dimensional stability for 3D-printed constructs.

### 3.3. Three Dimensionally Printed Plant-Based Protein

#### 3.3.1. Printing Performance of Printing Inks and Printing Accuracy

**Figure 7:**
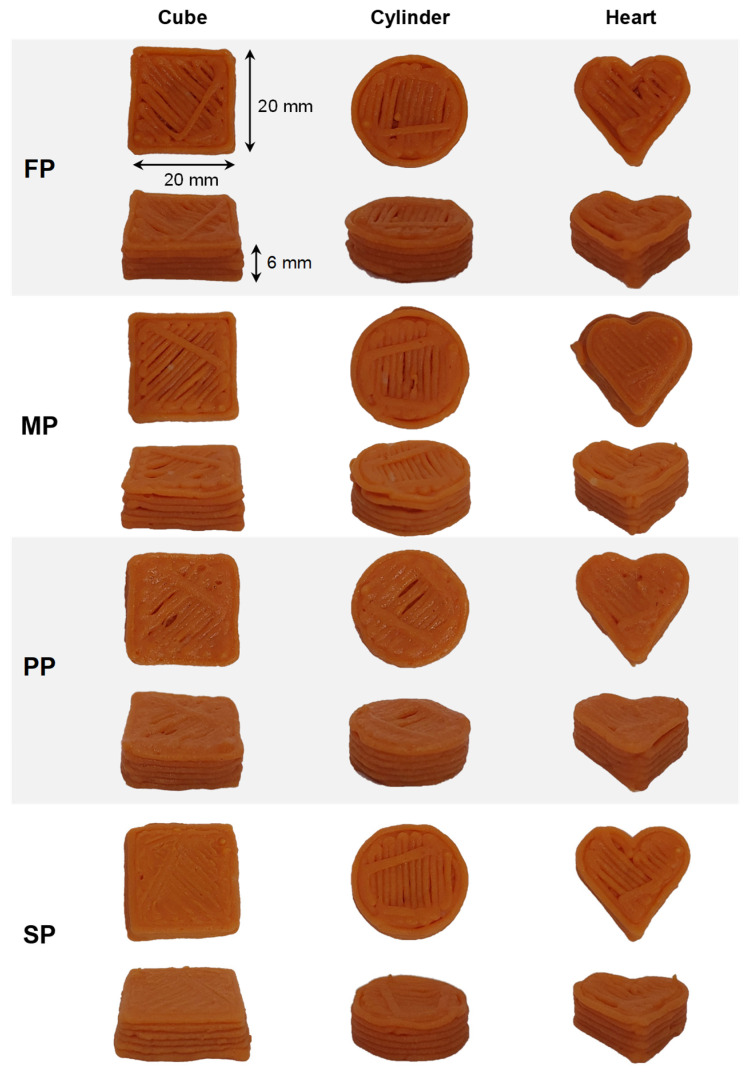
Printing results of the different plant-based inks containing 2.5% TGase powder (6.25 U/g plant-based protein) in various shapes.

Figure 7 presents morphological characteristics of 3D printed constructs. All TGase-crosslinked plant protein formulations, containing 20% plant-based protein content, 2.5% TGase powder (6.25 U/g plant-based protein), 1% xanthan gum, and 1% paprika oleoresin, were successfully extruded while maintaining the pre-designed geometries without structural collapse. Dimensional measurements were performed immediately after printing and after incubation at 55 °C for 2 h to allow TGase-mediated gelation. Dimensional deviations from CAD specifications were calculated for length, width, and height, and used as quantitative criteria for printability assessment (Table 3). The constructs exhibited well-defined edges, uniform layer deposition, and smooth surfaces, indicating enhanced viscoelastic properties and network cohesion. Consistent appearances across protein types demonstrated relatively uniform printing behavior, independent of botanical source. It is important to note that the observed printing performance likely reflects combined contributions from TGase-mediated crosslinking and the included additives. Xanthan gum enhances viscosity and shear-thinning behavior [13], while paprika oleoresin introduces minor hydrophobic interactions that may modulate protein network assembly. Thus, while TGase improves mechanical properties and printability [3], additive–protein interactions may also contribute to extrusion stability and structural integrity.

Printing accuracy of 3D-printed objects with TGase of 6.25 U/g plant-based protein demonstrated high dimensional fidelity across all formulations (Table 3). Length accuracy ranged from 93.2% (MP) to 95.9% (SP), width accuracy from 94.7–95.5%, and height accuracy from 95.7% (MP and SP) to 96.7% (PP). No statistically significant differences were observed among samples (*p* > 0.05), and small standard deviations indicate good process repeatability. Superior height accuracy reflects well-controlled extrusion and layer stacking, while slightly lower length accuracy in MP may result from minor material spreading during deposition. It should be noted that printability is a multifactorial property, influenced not only by dimensional fidelity but also by rheology, protein composition, and additive effects. Thus, the reported measurements provide one quantitative perspective on printability within the broader context of bioink performance for 3D food printing.

#### 3.3.2. Texture Profile Analysis

Texture profile analysis of 3D-printed plant protein gels with 2.5% TGase powder (6.25 U/g plant-based protein) revealed significant changes between immediate post-extrusion and after 2 h incubation at 55 °C (Table 3). Initial hardness values were relatively low (102.40–129.50 g), indicating limited structural development before crosslinking activation. After incubation, hardness increased markedly, ranging from 946.88 g (PP) to 1259.94 g (SP), demonstrating effective ε-(γ-glutamyl)lysine bond formation and protein matrix reinforcement [23]. Protein-specific differences were pronounced, with SP and FP achieving the highest post-incubation hardness while PP showed the lowest response, likely reflecting variations in lysine and glutamine accessibility for TGase activity. Similarly, Cheng et al. [13] reported that TGase incorporation enhanced structural integrity of 3D-printed constructs, increasing hardness and dimensional stability compared to controls. Adhesiveness dramatically reduced from highly negative values (−233.38 to −85.38 g·s) to near-neutral levels (−5.26 to −3.05 g·s), indicating enhanced structural integrity and reduced surface tackiness through crosslinked network development. SP demonstrated superior springiness (0.811 mm) and cohesiveness (0.300) compared to PP (0.607 mm and 0.256, respectively), critical parameters for meat-analog texture characteristics. Derived parameters followed similar trends, with SP exhibiting the highest chewiness (307.71) while gumminess showed no significant variation (*p* > 0.05). These results demonstrate TGase’s effectiveness in enhancing textural properties, with SP and FP showing superior crosslinking responsiveness. Similarly, Tay et al. [22] developed 3D-printed prawn mimics using fava bean proteins and found that TGase treatment enhanced chewiness from 818.8 to 940.6 g, with further improvement to 953.13 g after steaming. Differences correlate with amino acid composition and tertiary structure governing enzymatic substrate accessibility, highlighting the importance of protein source selection and TGase optimization for achieving target textural attributes in plant-based 3D food printing.

#### 3.3.3. Gel Microstructure

**Figure 8:**
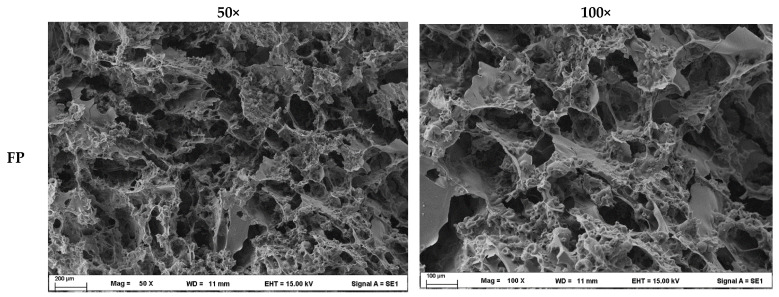

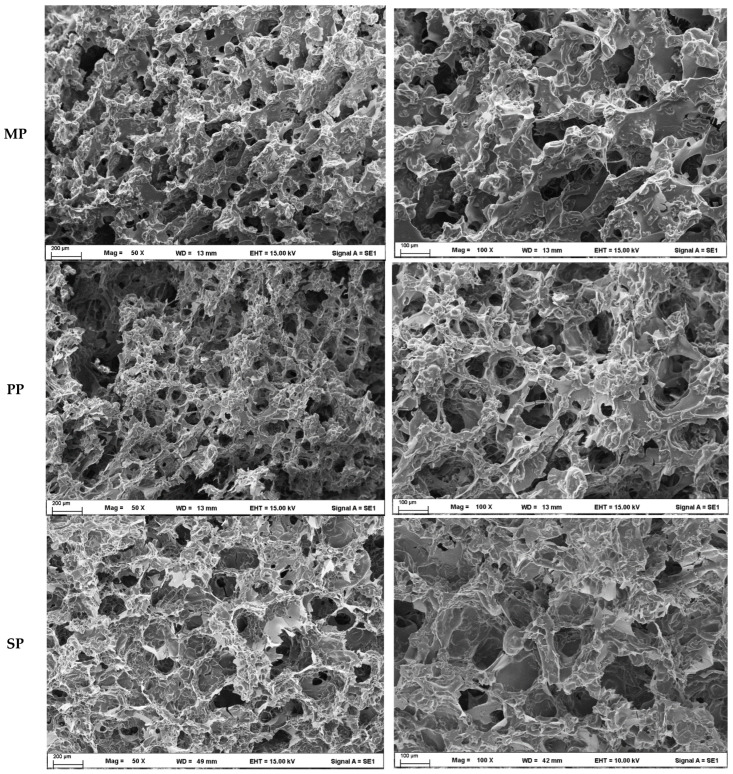
SEM microstructure of cross-sections of 3D printed plant proteins treated with 2.5% TGase powder (6.25 U/g plant-based protein) at 50× (**left**) and 100× (**right**) magnification. FP: fava bean protein isolate; MP: mung bean protein isolate; PP: pea protein isolate; SP: soy bean protein isolate.

SEM analysis of plant protein gels treated with TGase revealed substantial structural changes associated with enzymatic crosslinking (Figure 8). Network structures varied among protein sources: SP gels exhibited homogeneous, compact, and continuous matrices, whereas PP gels formed large, irregular aggregates with porous, discontinuous structures due to weak protein–protein interactions. These loose networks corresponded to poor texture, mechanical strength, and water-holding capacity typical of heat-induced gels lacking sufficient crosslinking. We note that the SEM images presented (50× and 100× magnification) provide an overview of surface morphology and gross structural differences rather than detailed network architecture, higher magnification would be required to directly visualize fine fibrillar networks formed by TGase crosslinking. These experimental findings corroborate the TPA analysis, wherein SP formulations exhibited superior hardness, springiness, and chewiness values, corresponding to enhanced crosslinking density. Consistent with Yu et al. [64], TGase crosslinking of pre-denatured SP produced more compact and uniform microstructures with regular pores and orderly arrangement compared to native matrices. Cheng et al. [13] also observed that samples treated with TGase exhibited a reticular cross-linked structure displaying the most pronounced flake-like morphology. Enzymatic ε-(γ-glutamyl)lysine bond formation enhanced intermolecular interactions, generating denser networks with smaller, uniform aggregates that improved gel firmness. Functionally, these structural changes correlate with rheological behavior, where SP exhibited higher G′ and lower tan δ values than other plant proteins, reflecting a more solid-like gel suitable for 3D printing applications. The combination of extensive antiparallel β-sheet formation and increased backbone packing explains SP’s superior texture, shape fidelity in printed constructs. Compared to other plant proteins, which showed less pronounced β-sheet enrichment and retained porous, discontinuous microstructures under SEM, SP’s dense and uniform networks highlight its suitability for precision extrusion-based printing. Although microstructural patterns differed among proteins due to compositional and reactivity variations, TGase consistently increased network compactness and uniformity. We emphasize that the conclusions regarding network formation and protein gelation are supported by complementary TNBS, NMR, and SR-FTIR analyses, rather than solely by SEM images. SEM evidence confirms TGase’s pivotal role in reinforcing plant protein networks, supporting improvements in macroscopic texture and demonstrating its utility for tailoring functional properties in plant-based gels.

## 4. Conclusions

TGase can enhance the functionality of plant-based proteins for 3D food printing, although the overall performance of bioinks is influenced not only by enzymatic crosslinking but also by formulation additives and carrier proteins present in commercial TGase preparations. TGase-mediated treatment promoted protein polymerization and structural modifications, including α-helix to β-sheet transitions, contributing to stronger and more cohesive networks. Rheological analyses indicated improved viscoelasticity and gelation behavior, supporting shape fidelity and stability during extrusion. Among the proteins tested, SP and FP exhibited particularly favorable texture and mechanical performance, as reflected by TPA and SEM analyses, where denser and more uniform microstructures were observed. These findings suggest that TGase, in combination with appropriate formulation strategies, can be used to optimize plant-based bioinks for enhanced printability, structural integrity, and textural quality. The study provides guidance for the development of high-quality plant-based gels and meat analogs, while highlighting the importance of considering both enzymatic activity and formulation design in 3D food printing applications.

## Figures and Tables

**Table 1:** Reduction in accessible primary amines in plant-based proteins treated with different TGase concentrations, determined by TNBS assay.

Sample	TGase Concentration (U/g Plant Protein)	Reduction in Accessible Primary Amines (%)
FP	10	27.46 ± 3.57 ^aA^
	20	36.79 ± 3.79 ^aB^
	50	55.69 ± 1.34 ^aC^
MP	10	29.85 ± 0.98 ^abA^
	20	44.70 ± 1.15 ^bB^
	50	56.26 ± 5.22 ^aC^
PP	10	30.53 ± 3.72 ^abA^
	20	45.90 ± 3.63 ^bB^
	50	62.34 ± 3.60 ^abC^
SP	10	38.63 ± 1.47 ^cA^
	20	50.41 ± 1.68 ^bB^
	50	64.57 ± 1.55 ^bC^

Note: Mean ± SD from quadruplicate determinations. FP: fava bean protein isolate; MP: mung bean protein isolate; PP: pea protein isolate; SP: soy protein isolate. Different lowercase letters indicate significant differences between samples at the same TGase concentration (*p* < 0.05). Different uppercase letters indicate significant differences among TGase concentration treatments within the same plant-based protein sample (*p* < 0.05).

**Table 2:** Relative content (%) of protein secondary structures determined from the amide I region of the synchrotron-FTIR spectrum (after curve fitting).

Protein Secondary Structures (%)	*α*-Helix (1640–1670 cm^−1^)	*β*-Turn (1670, 1620 cm^−1^)	*β*-Sheet (1620–1640 cm^−1^)	Antiparallel (1680–1695 cm^−1^)
FP	36.43 ± 0.54 ^a^	22.83 ± 1.01	20.32 ± 0.42	20.42 ± 0.38 ^b^
FP-C	35.05 ± 0.25 ^b^	21.62 ± 0.40	21.49 ± 0.80	21.84 ± 0.51 ^a^
MP	33.78 ± 1.01	22.28 ± 0.36	22.45 ± 0.60	21.06 ± 0.55 ^b^
MP-C	32.06 ± 0.93	22.18 ± 0.44	22.88 ± 0.54	23.31 ± 0.50 ^a^
PP	35.60 ± 0.22 ^a^	25.45 ± 0.49 ^a^	16.92 ± 1.23 ^b^	22.03 ± 0.70
PP-C	31.19 ± 0.40 ^b^	22.24 ± 0.52 ^b^	24.39 ± 0.50 ^a^	22.18 ± 0.46
SP	31.04 ± 0.58	24.74 ± 0.79 ^a^	23.37 ± 1.04	21.12 ± 1.02 ^b^
SP-C	30.77 ± 0.34	21.28 ± 0.34 ^b^	24.04 ± 0.53	23.64 ± 0.24 ^a^

Different lowercase letters indicate significant differences between samples with and without crosslinking (*p* < 0.05). The absence of lowercase letters indicates no significant difference (*p* > 0.05).

**Table 3:** Printing accuracy and textural properties of 3D-printed plant-based protein crosslinked with TGase (6.25 U/g protein).

Parameters	FP	MP	PP	SP
Printing accuracy (%)				
Accuracy Length ^ns^	95.3 ± 2.1	93.2 ± 1.6	94.2 ± 2.0	95.9 ± 1.7
Accuracy Width ^ns^	94.7 ± 1.5	95.5 ± 1.8	95.4 ± 1.9	95.2 ± 2.0
Accuracy Height ^ns^	96.3 ± 3.0	95.7 ± 3.2	96.7 ± 3.1	95.7 ± 2.2
Texture properties				
After printing				
Hardness (g)	116.238 ± 13.444 ^abc^	120.398 ± 15.706 ^bc^	102.398 ± 15.935 ^a^	129.498 ± 13.851 ^c^
Adhesiveness (g·s)	−103.303 ± 30.686 ^c^	−162.876 ± 42.969 ^b^	−233.376 ± 28.424 ^a^	−85.376 ± 26.124 ^c^
After incubation				
Hardness (g)	1202.933 ± 118.759 ^b^	1200.118 ± 177.217 ^b^	946.884 ± 157.208 ^a^	1259.942 ± 162.222 ^b^
Adhesiveness (g·s)	−3.510 ± 1.276 ^ab^	−4.103 ± 2.375 ^ab^	−5.256 ± 1.804 ^a^	−3.050 ± 1.576 ^b^
Springiness (mm)	0.664 ± 0.086 ^a^	0.679 ± 0.079 ^a^	0.607 ± 0.084 ^a^	0.811 ± 0.052 ^b^
Cohesiveness	0.267 ± 0.024 ^a^	0.279 ± 0.018 ^a^	0.256 ± 0.032 ^a^	0.300 ± 0.044 ^b^
Gumminess ^ns^	344.152 ± 60.090	325.352 ± 72.815	301.952 ± 85.206	379.952 ± 86.317
Chewiness	252.620 ± 62.015 ^a^	242.788 ± 37.710 ^a^	219.714 ± 72.139 ^a^	307.714 ± 71.843 ^b^

Different lowercase letters indicate significant differences between samples with and without crosslinking (*p* < 0.05). ns: The absence of lowercase letters indicates no significant difference (*p* > 0.05).

## Data Availability

The original contributions presented in this study are included in the article. Further inquiries can be directed to the corresponding author.

## Supplementary Materials

The following supporting information can be downloaded at: https://www.mdpi.com/article/10.3390/foods15020322/s1, Figure S1: The curve fitting of amide I and secondary structure protein band assignment (1700–1600 cm^−1^) of plant proteins; Figure S2: Loading plot of plant proteins with and without T-Gase; (A) fava bean, (B) mung bean, (C) pea, and (D) soybean; Figure S3: Correlation loading plot (B) of plant proteins with and without T-Gase; (A) fava bean, (B) mung bean, (C) pea, and (D) soybean; Figure S4: Printing results of the soy protein isolate-based inks containing different concentration TGase (2.5, 6.25, 12.5 U/g plant-based protein) in various shapes. Table S1: Metabolites identified by ^1^H-NMR in TGase-crosslinked plant proteins [66,67,68,69,70,71,72].

## Abbreviations

The following abbreviations are used in this manuscript:

FP	Fava bean protein isolate
MP	Mung bean protein isolate
PP	Pea protein isolate
SP	Soy bean protein isolate
TGase	Transglutaminase
