# Supplementary material for: Plant-Based Protein Bioinks with Transglutaminase Crosslinking: 3D Printability and Molecular Insights from NMR and Synchrotron-FTIR
Source: Foods. 2026 Jan 15;15(2):322. doi: 10.3390/foods15020322 (PMC12841553; doi:10.3390/foods15020322)
Supplement: Supplementary file 1 [file foods-15-00322-s001.zip › foods-4088809-supplementary.pdf]

**Supplementary Figures and Tables**

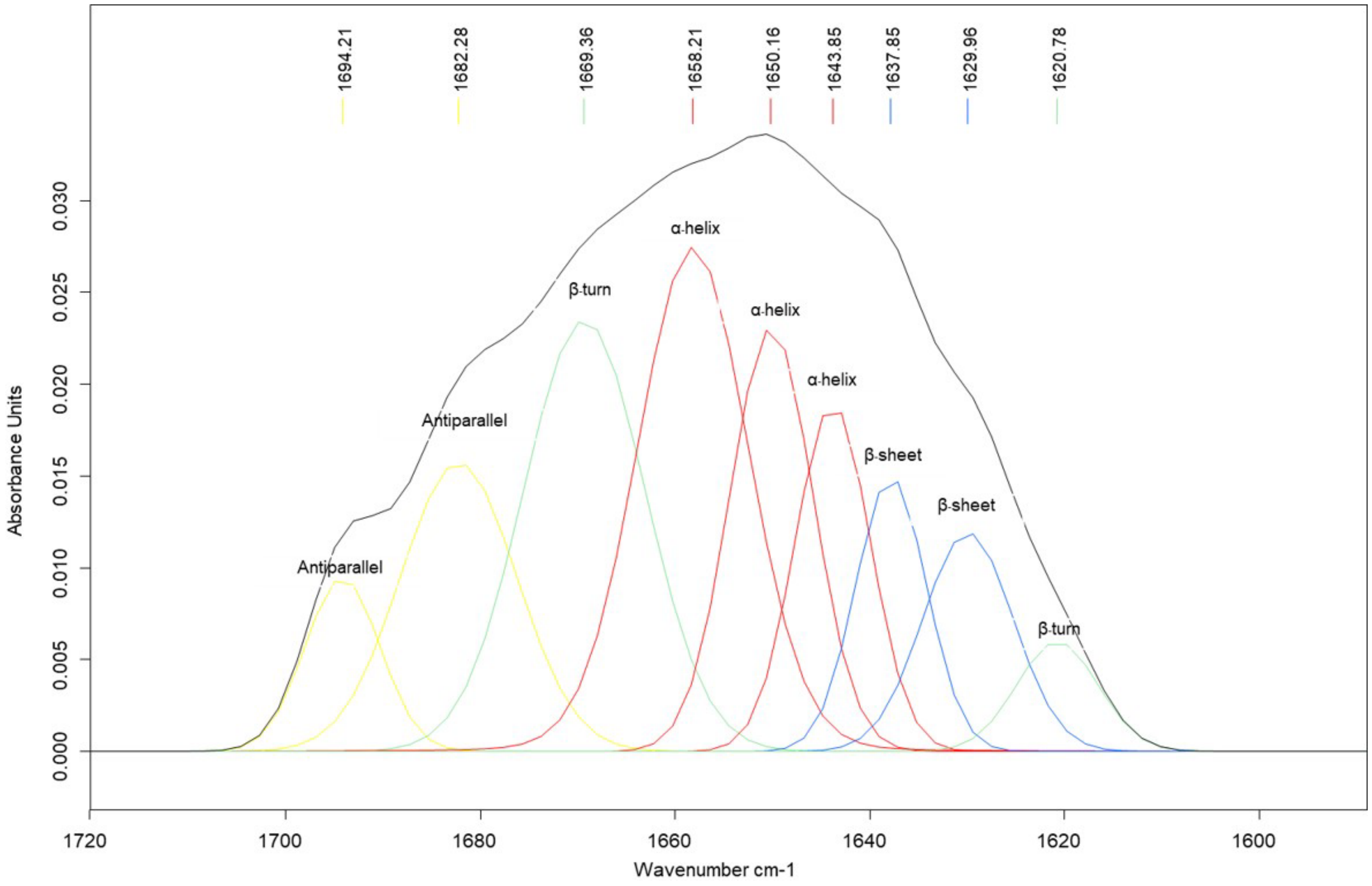


**Figure S1.** The curve fitting of amide I and secondary structure protein band assignment (1700-1600 $cm^{-1}$) of plant proteins.

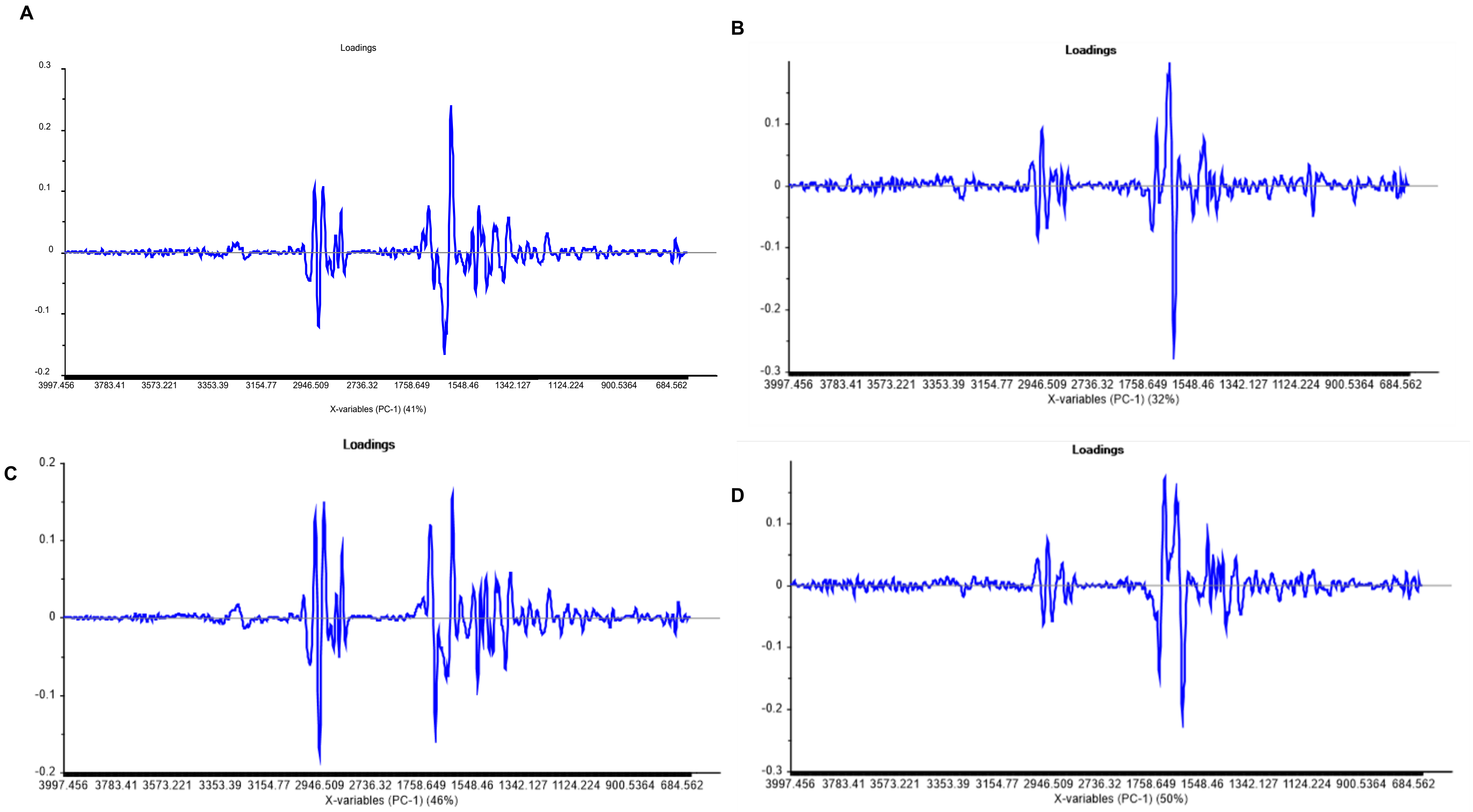


**Figure S2.** Loading plot of plant proteins with and without T-Gase; (A) fava bean, (B) mung bean, (C) pea, and (D) soybean.

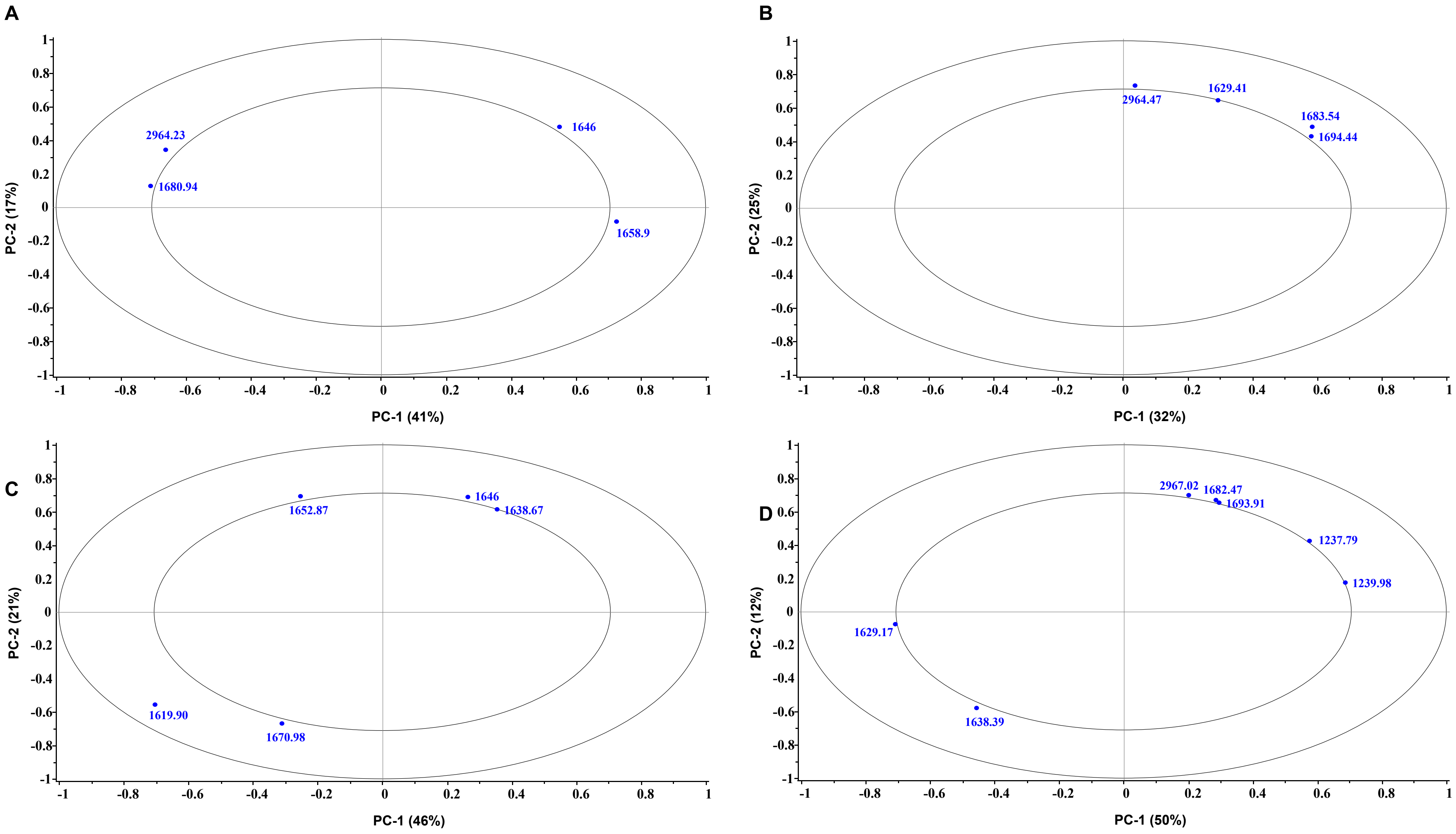


**Figure S3.** Correlation loading plot (B) of plant proteins with and without T-Gase; (A) fava bean, (B) mung bean, (C) pea, and (D) soybean.

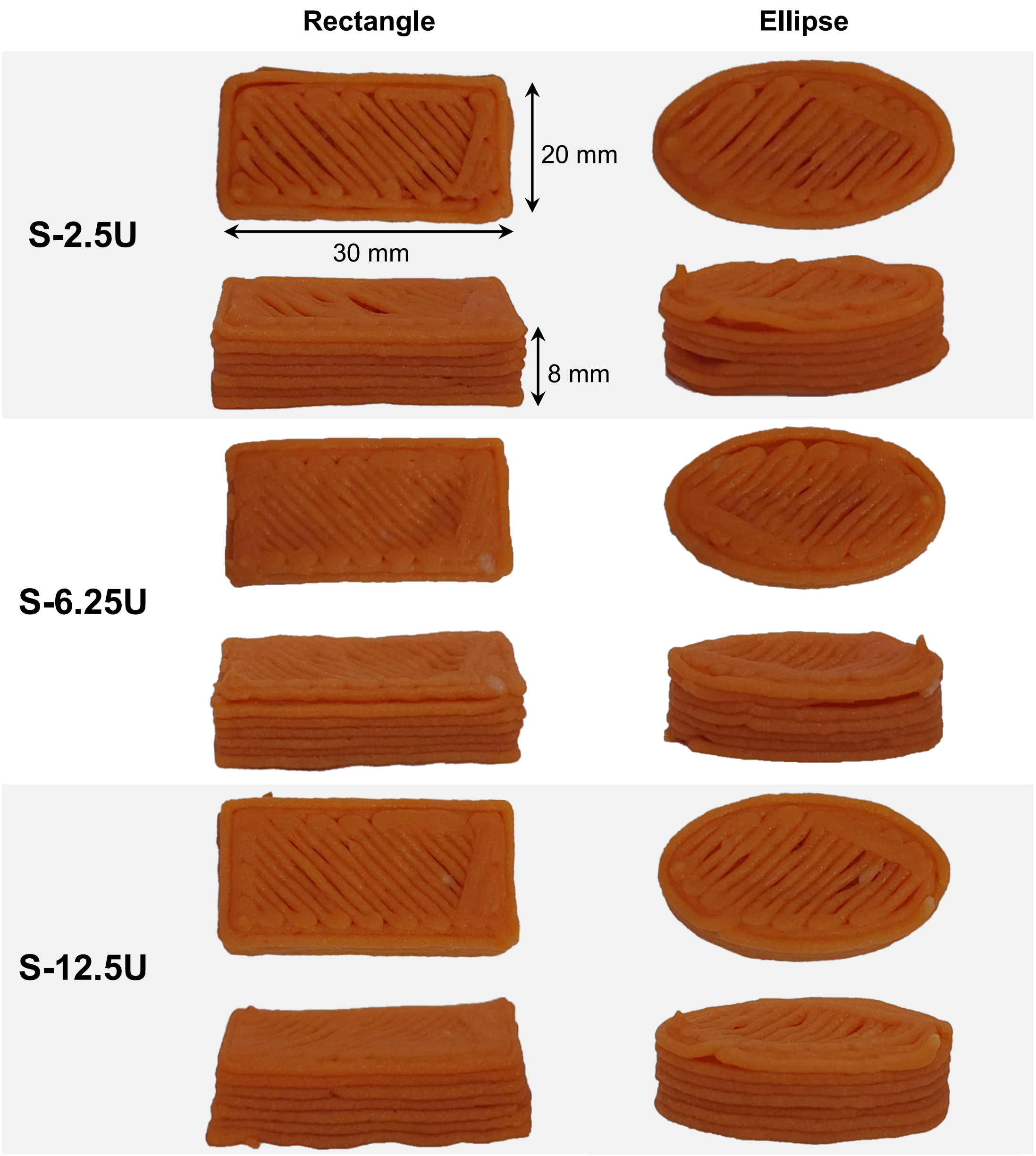


**Figure S4.** Printing results of the soy protein isolate-based inks containing diferent concentration TGase (2.5, 6.25, 12.5U/g plant-based protein) in various shapes.

**Table S1:** Metabolites identified by $^{1}H$ NMR in TGase-crosslinked plant proteins.

| Number | Metabolites | Multiplicity | | References |
|---|---|---|---|---|
| | | This Study | References | |
| 1 | Valine | 0.93(d), 1.16(d), 2.23(m), 3.58(d) | 0.98(d), 1.03(d), 2.26(m), 3.60(d) | HMDB, Ref. [66–69] |
| 2 | Lactate | 1.34(d), 4.13(q) | 1.32(d), 4.10(q) | HMDB, Ref. [66–69] |
| 3 | Methionine | 2.18(m), 2.66(broad), 3.83(d) | 2.16(m), 2.63(t), 3.85(dd) | HMDB, Ref. [66–69] |
| 4 | Succinate | 2.41(s) | 2.39(s) | HMDB, Ref. [66–69] |
| 5 | *O*-phosphocholine | 3.23(s) | 3.22(s) | Ref. [70] |
| 6 | Betaine | 3.27(s), 3.91(s) | 3.25(s), 3.89(s) | HMDB, Refs. [66–69] |
| 7 | Glucose | 5.25(d) | 5.22(d) | Ref. [71] |
| 8 | Inosine | 4.30(dd), 4.43(dd, 6.09(d), 8.21(s), 8.35(s) | 4.26(dd), 4.42(dd), 4.80(s), 6.05(d), 8.19(s), 8.30(s) | HMDB, Ref. [66–69] |
| 9 | Adenosine monophosphate | 4.52(t), 6.11(d), 8.14(s), 8.58(s) | 4.62(t), 4.78(m), 6.10(d), 8.18(s), 8.49(s) | HMDB, Ref. [66–69] |
| 10 | Nicotinate | 7.48(m), 8.21(s), 8.84(s), 9.13(s) | 7.50(t), 8.23(s), 8.59(s), 8.93(s) | HMDB, Ref. [66–69] |
| 11 | 3,4-Dihydroxybenzeneacetate | 3.41(s), 6.78(d), 6.83(d), 6.88(broad) | 6.88(d), 6.82(d), 6.72(dd), 3.40(s) | Ref. [72] |
| 12 | Gallate | 7.09(s) | 7.04 (s) | Ref. [72] |
| 13 | Alanine | 1.47(d), 3.76(q) | 1.47(d), 3.77(q) | HMDB, Ref. [66–69] |
| 14 | Galactarate | 3.25(m), 3.39(broad), 3.48(m), 4.27(s) | 3.22(dd), 3.38(m), 3.48(m), 4.26(s) | Ref. [71] |
| 15 | Methyl soyate | 1.55(m) | 1.59(m) | Ref. [70] |
| 16 | Malonate | 3.13(s) | 3.10(s) | Ref. [70] |
| 17 | Choline | 3.19(s), 3.51(d), 4.04(m) | 3.19(s), 3.51(dd), 4.06(m) | HMDB, Ref. [66–69] |
| 18 | Diazin | 7.25(d) | 7.24(d) | HMDB |

References

66. Shen, G.; Huang, Y.; Dong, J.; Wang, X.; Cheng, K.-K.; Feng, J.; Xu, J.; Ye, J. Metabolic effect of dietary taurine supplementation on Nile tilapia (*Oreochromis niloticus*) evaluated by NMR-based metabolomics. *J. Agric. Food Chem.* **2018**, *66*, 368–377. https://doi.org/10.1021/acs.jafc.7b03182.
67. Shen, G.P.; Ding, Z.N.; Dai, T.; Feng, J.H.; Dong, J.Y.; Xia, F.; et al. Effect of dietary taurine supplementation on metabolome variation in plasma of Nile tilapia. *Animal* **2021**, *15*, 100167. https://doi.org/10.1016/j.animal.2020.100167.
68. Schock, T.B.; Newton, S.; Brenkert, K.; Leffler, J.; Bearden, D.W. An NMR-based metabolomic assessment of cultured cobia health in response to dietary manipulation. *Food Chem.* **2012**, *133*, 90–101.
69. Allen, P.J.; Wise, D.; Greenway, T.; Khoo, L.; Griffin, M.J.; Jablonsky, M. Using 1D $^1$H and 2D $^1$H J-resolved NMR metabolomics to understand the effects of anemia in channel catfish (*Ictalurus punctatus*). *Metabolomics* **2015**, *11*, 1131–1143. https://doi.org/10.1007/s11306-014-0767-2.
70. Ethier, R.; Krishnamurthy, A.; Jeffrey, M.; Tompkins, T.A. Profiling of metabolites in a fermented soy dietary supplement reinforces its role in the management of intestinal inflammation. *Mol. Nutr. Food Res.* **2024**, *68*, 2300770. https://doi.org/10.1002/mnfr.202300770.
71. Zhou, Y.; Kim, S.-Y.; Lee, J.-S.; Shin, B.-K.; Seo, J.-A.; Kim, Y.-S.; Lee, D.-Y.; Choi, H.-K. Discrimination of the geographical origin of soybeans using NMR-based metabolomics. *Foods* **2021**, *10*, 435. https://doi.org/10.3390/foods10020435.
72. Correia, B.S.B.; Bertram, H.C.; Sørensen, E.B.; Aaslyng, M.D. Metabolome of different cultivars of peas, lentils, faba beans and lupins—An ^1H NMR spectroscopic exploration of their sensory attributes and potential biofunctionality. *Food Chem.* **2025**, *477*, 143579. https://doi.org/10.1016/j.foodchem.2024.143579.
